# Pediculosis Capitis with Id Reaction and Plica Polonica

**DOI:** 10.4269/ajtmh.21-0271

**Published:** 2021-07-12

**Authors:** Anusuya Sadhasivamohan, Kaliaperumal Karthikeyan, Vijayasankar Palaniappan

**Affiliations:** Department of Dermatology, Venereology and Leprosy, Sri Manakula Vinayagar Medical College and Hospital, Puducherry, India

**Figure 1.**
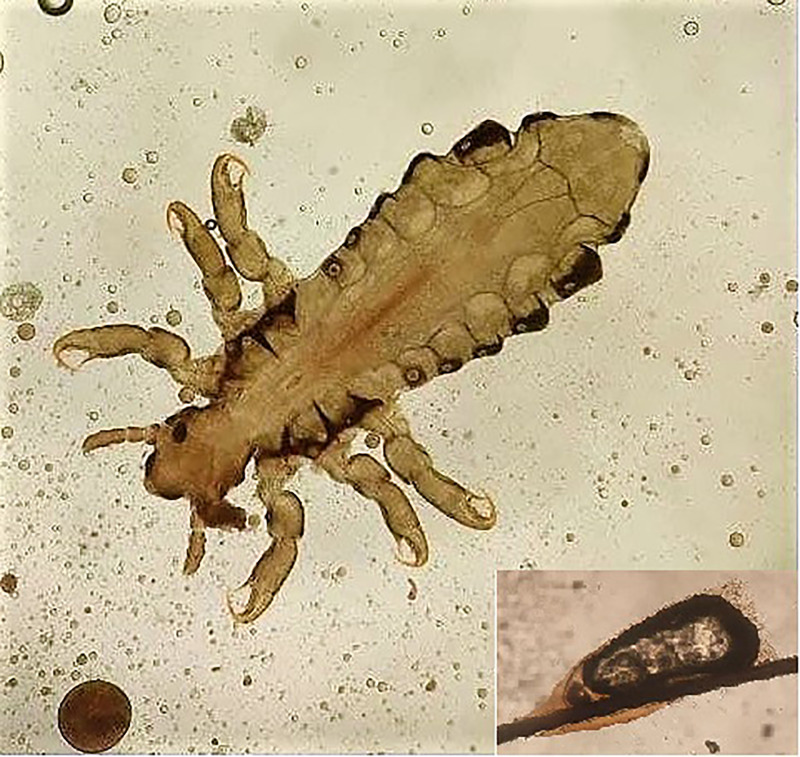
Innumerable nits with serous crusts and right ear eczema. Id reaction over the nape of neck. This figure appears in color at www.ajtmh.org.

**Figure 2.**
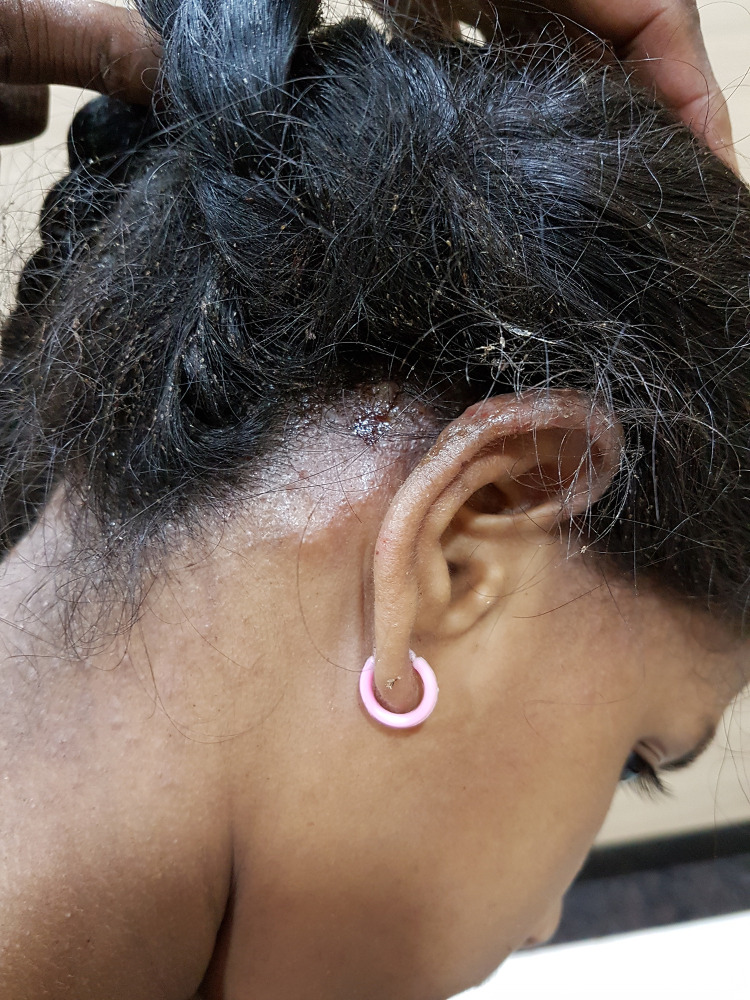
Microscopic view of the louse with nit (inset). This figure appears in color at www.ajtmh.org.

A 9-year-old girl from a low socioeconomic condition presented with severe itchy scalp for 6 months and oozy lesions over ears bilaterally for 3 weeks. Scalp examination showed innumerable nits, lice with scales, and serous crusts. She also had tender, erythematous, oozy, eczematous plaques, both over and behind the earlobes, associated with tender occipital lymphadenopathy. A few skin-colored papules were noted over the nape of neck (Figure 1). Rest of the physical examination was normal. Her hemogram showed iron deficiency anemia (Hb 7.0 g/dL). A clinical diagnosis of severe pediculosis capitis infestation with Id reaction and eczematous dermatitis was made. A louse was retrieved and viewed under the microscope (Figure 2, inset). She was given a course of 1% permethrin rinse (weekly once) for 2 weeks, two doses of ivermectin (6 mg 1 week apart), and topical steroids for the ear eczema. Four weeks later, she was symptom free. She was also educated on hygiene practices to avoid further infestation.

Pediculosis capitis, caused by *Pediculus humanus capitis*, is most common in 3- to 12-year-old female children.^1^^,^^2^ It is usually transmitted by close personal contact and through fomites.^2^ The louse saliva antigen elicits an inflammatory response that causes pruritus and scratching, resulting in secondary impetignization.^2^ Id reaction is an autosensitization dermatitis that manifests as itchy, erythematous, maculopapular/papulovesicular lesions distant from the primary inflammatory focus. The Id reaction secondary to pediculosis is called pediculid.^3^ Florid pediculosis infestation can also lead to fever, malaise, cervical and occipital lymphadenopathy, iron deficiency anemia, and plica polonica.^4^ Plica polonica is a compact mass of scalp hair with irreversibly entangled plaits stuck together with exudate and dirt.^5^ The diagnosis is based on clinical features and the management of pediculosis is with topical 1% permethrin and oral ivermectin. Other treatment modalities include wet combing, 0.5% malathion, and newer drugs such as spinosad. The pediculid and eczema will eventually subside once pediculosis is treated. Sometimes it may require a short course combined topical antibiotic and steroid.^4^

This case illustrates the importance to promptly diagnose and treat pediculosis capitis to prevent severe infestation and its sequelae.
